# The complete mitochondrial genome of *Celypha flavipalpana* (Lepidoptera: Tortricidae) from Southeast Tibet of China

**DOI:** 10.1080/23802359.2020.1829129

**Published:** 2020-10-09

**Authors:** Dong Xiang

**Affiliations:** Institute of Vegetable, Tibet Academy of Agricultural and Animal Husbandry Sciences, Lhasa, China

**Keywords:** *Celypha flavipalpana*, Mitogenome, next-generation sequencing, phylogeny

## Abstract

The complete mitogenome of *Celypha flavipalpana* (Lepidoptera: Tortricidae) was sequenced, assembled and annotated. The genome has a circular genome of 15,691bp in length, with 13 protein-coding genes (PCGs), 22 tRNAs, 2 rRNAs, and a control region. Phylogenetic analysis revealed that Trichinidae is a monophyletic group and *Celypha* (*C. flavipalpana*) as sister of the genus *Phiaris* (*P. dolosana*).

Trichinidae is a large family of Lepidoptera, and currently consist of more than 9000 known species worldwide, and 558 of which have been recorded from China hitherto (Brown, 2005; Liu and Li 2002). Pests of Trichinidae can cause significant economic losses to agriculture, forestry and horticulture production in temperate regions (Jia et al. 2010). To date, a total of 47 genera and 100 species of this family have been recorded from the Qinghai-Tibet Plateau (Zhang and Wang 2016). *Celypha* Hübner is an important genus in Trichinidae, and consisting of three pest species, and only two of them were recorded in Qinghai Province, but not in Tibet, China (Zhang and Wang 2016). The known hosts of *Celypha* species are thyme plants, and they are also potential pests for other crops (Liu and Li 2002). For example, *Celypha flavipalpana* was found as a new pest for maize seedling stage in Fuxin City, Liaoning Province, China, and can lead to growing weak and even dying of the seedlings (Wang et al. 2013). However, current researches are only focus on the morphological identification and geographic distribution of *Celypha* species (Wang and Wang 2015), their phylogenetic relationships are poorly understood, especially in the whole mitogenome data based on next generation sequencing.

In this study, the adult specimens of *C. flavipalpana* were collected from the orchard of Baxoi County in Tibet, China (30°02′ 67.73″ N, 94°02′ 41.44″ E). Specimen (Voucher No. 20190708-05) was deposited in Tibet Academy of Agricultural and Animal Husbandry Sciences. The total genomic DNA was extracted from the body by a traditional phenol-chloroform method (Sambrook and Russell 2001). After DNA isolation, 1 μg of purified DNA was fragmented and used to construct short-insert libraries (insert size 430 bp) according to the manufacturer’s instructions (Illumina), then sequenced on the Illumina Hiseq 4000 (Borgstrom et al. 2011). The mitochondria genome was reconstructed using a combination of de novo and reference-guided assemblies, and the following three steps were used to assemble mitochondria genomes. First, the filtered reads were assembled into contigs using SOAPdenovo 2.04 (Li et al. 2010). Second, contigs were aligned to the reference genome of species using BLAST, and aligned contigs (≥80% similarity and query coverage) were ordered according to the reference genome. Third, clean reads were mapped to the assembled draft mitochondria genome to correct the wrong bases, and the gaps were filled through local assembly. The transfer RNA (tRNA) genes were identified and then the secondary structures of tRNAs were predicted using tRNAscan-se (Lowe and Chan 2016).

The mitogenome of *C. flavipalpana* is a circular molecule of 14,979 bp in size (Genbank accession number is MT548574), including 13 proteincoding genes (PCGs), 22 transfer RNA (tRNA) genes, 2 ribosomal RNA (rRNA) genes and one control region. The *C. flavipalpana* mitogenome has an A–T content of 80.8% (40.6% of A, 40.2% of T, 11.4% of C, and 7.8% of G), which is a typical structure of Lepidopterans mitogenome. No gene arrangement was found in the sequence of *C. flavipalpana* mitogenome, which has the similar characteristic with other Lepidopterans. The light strand encoded 9 PCGs (*nad2, co1, co2, atp8, atp6, co3, nad3, nad6, and cytb*), and the other four (*nad1, nad4, nad4l, and nad5*) were on the heavy strand. All tRNA genes are identified by MITOS web server (Bernt et al. 2013), and are varied from 64 bp (*trnT* and *trnA*) to 79 bp (*trnK*) in length. The length of 16S rRNA and 12S rRNA were 1,398bp and 781 bp, respectively.

Phylogenetic tree (Figure 1) was constructed based on the whole mitogenome sequence of 14 Tortricidae species and one Bombycidae species (*Bombyx mandarina*) as outgroup using the Maximum Likelihood (ML) method with 1000 bootstrap replicates using MEGA 7.0 (Kumar et al. 2016). The monophyly of Tortricidae was strongly supported by ML tree based on the whole mitogenome data, and *Celypha* (*C. flavipalpana*) is sister of the genus *Phiaris* (*P. dolosana*) with high support values.

**Figure 1.**
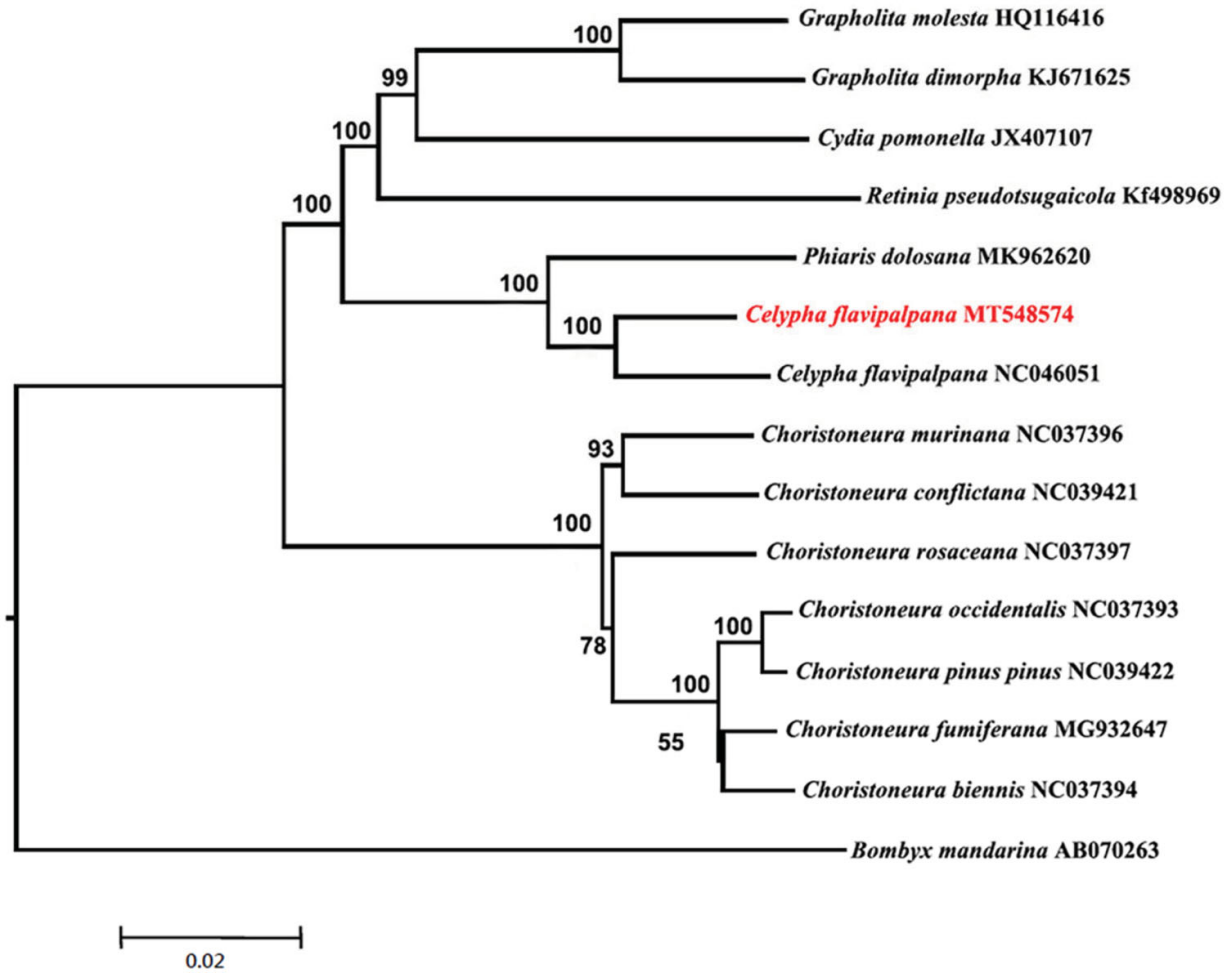
Maximum-Likelihood phylogenetic tree of 14 Tortricidae species including *C. flavipalpana* based on 15,691 bp of 13 concatenated PCGs. Numbers at nodes are bootstrap values from 1000 replicates.

## Data Availability

The data that support the findings of this study are openly available in GenBank of NCBI at https://www.ncbi.nlm.nih.gov, reference number MT548574.1
